# Facial Expression Recognition in Children with Cochlear Implants and Hearing Aids

**DOI:** 10.3389/fpsyg.2016.01989

**Published:** 2016-12-21

**Authors:** Yifang Wang, Yanjie Su, Song Yan

**Affiliations:** ^1^Beijing Key Laboratory of Learning and Cognition, Department of Psychology, Capital Normal UniversityBeijing, China; ^2^Department of Psychology, Peking UniversityBeijing, China; ^3^School of Humanities and Social Sciences, Jacobs UniversityBremen, Germany

**Keywords:** facial expression recognition, cochlear implants, hearing aids, verbal task, non-verbal task

## Abstract

Facial expression recognition (FER) is an important aspect of effective interpersonal communication. In order to explore whether the development of FER was delayed in hearing impaired children, 44 child participants completed labeling, and matching tasks to identify four basic emotions (happiness, sadness, anger, and fear). Twenty-two participants had either a cochlear implant (CI) or a hearing aid (HA) while 22 had normal hearing and participants were matched across conditions by age and gender. The results showed that children with a CI or HA were developmentally delayed not only in their emotion-labeling (verbal) tasks but also in their emotion-matching (nonverbal) tasks. For all participants, the emotion-labeling task was more difficult than the emotion-matching task. Additionally, the relative difficulty of recognizing four different emotional expressions was similar between verbal and nonverbal tasks.

## Introduction

Facial expression recognition (FER) is important for social interactions and effective communication. Deficits in young children’s ability to recognize facial expressions can lead to impairments in social functioning (Herba and Phillips, 2004; Batty and Taylor, 2006). Denham et al. (1990) showed that peer-rated popularity and academic achievement correlated strongly with the ability to recognize others’ emotional expressions.

Hiroko and Yamaguchi (2014) found that Japanese babies between the age of 6 and 7 months were highly sensitive to angry facial expressions. This could possibly be an adaptation that might allow them to determine if they are in potentially dangerous situations. Infants also use emotional expressions as behavioral cues. For example, when their mothers appeared happy, they were more likely to participate in novel situations. Developmental researchers used different paradigms (matching and labeling) to measure how accurately children recognized facial expressions of different emotions (Markham and Adams, 1992; Bruce et al., 2000). They found that, compared to children who were 3 to 6 years old, older children more accurately recognized facial expressions (Denham et al., 1990; Widen and Russell, 2008).

In China, there are 115,000 children under 7 years old with severe to profound or complete deafness and 30,000 babies are born with hearing impairments annually (Liang and Mason, 2013). A cochlear implant (CI) is a device that provides direct electrical stimulation to the auditory nerve in the inner ear, giving deaf individuals the ability to hear. Children with severe to profound hearing loss (71 and 90 dB HL or greater) who cannot be helped with hearing aids (HI) may resort to CIs.

Some researchers have examined the broader effects of a CI or HA on children’s emotional and social development. Ziv et al. (2013) investigated the FER of hearing children, deaf children who communicated with sign language, and children with a CI who communicated orally. They found that when completing labeling tasks, no significant difference in performance existed among the three groups. Additionally, they found that in pointing tests, children with a CI and those in the hearing group achieved higher scores than deaf children who could communicate using sign language. Finally, they found no significant difference between children with a CI and children with normal hearing. However, Wang et al. (2011) found that children with a CI or HA displayed less developed FER compared to children with normal hearing, especially regarding their ability to recognize anger and fear. Additional support for Wang, Su, Fang, and Zhou’s finding was found by Wiefferink et al. (2013). Their findings revealed that compared to hearing children, children between the ages of 2.5 and 5 years old with a CI were less proficient in emotion recognition of facial expressions. In all three studies, children were presented with four photos of facial expressions and then randomly asked “Who looks happy/sad/angry/fearful”. The respective image that the children indicated was recorded in another study, Most and Aviner (2009) used a written emotion vocabulary test containing 36 items to examine 40 children between the ages of 10 and 17 years. Their sample included children with normal hearing, children with a CI, and children with a HA. Each item was designed to trigger a specific emotion. The participants were asked to indicate if each facial expression showed happiness, sadness, anger, or fear. The children in the study were matched by age and gender and the results showed that children with normal hearing were no more proficient at FER than children with a CI or HA. Additionally, no differences were found between children with a CI and HA. Moreover, Hopyan-Misakyan et al. (2009) found similar results in a study of children with a CI who were between 7 and 13 years old.

McClure (2000) asserted that there are two phases of FER development. The first is the ability to discriminate between different facial expressions, independent of language skills. The matching task mentioned above is a demonstration of this initial stage of FER. In this task, children had to match the emotional expressions of persons in one group to those of persons in another group purely based off the visual stimuli presented. Subsequently, researchers found amygdala activation during these types of non-verbal FER tasks with low cognitive demands (Herba et al., 2006). Another study involved identifying and labeling facial expressions. Children were asked to point to the facial expression that matched the label. Attenuated amygdala activation and increased prefrontal activation were observed during the verbal FER tasks (Phillips et al., 2003).

Székely et al. (2011) found that normally developing three year olds showed a difference in the levels of recognition of the four basic emotions (happiness, sadness, anger, and fear) between the verbal and nonverbal FER tasks. For example, on the nonverbal (emotion-matching) task, fear was most easily recognized, while on the verbal (emotion-labeling) task, fear was the most difficult to recognize. It is possible that in the early rehabilitation of children with a CI or HA, the nonverbal (e.g., matching) task was more suitable.

The primary purpose of the present study was to explore the differences between the performance of children with a CI or HA and normal children, who were matched by age and gender, for emotion-matching (nonverbal) tasks and emotion-labeling (verbal) tasks. The secondary purpose was to examine which FER task was more difficult and which emotional expressions (happiness, sadness, anger, and fear) were the most difficult to recognize during the verbal and nonverbal tasks. We assumed that children with a CI or HA in the early rehabilitation were developmentally delayed for both emotion-matching and emotion-labeling tasks.

## Materials and Methods

### Participants

The experiment included 22 children with a CI or HA (13 boys and 9 girls) from Beijing Sullivan Rehabilitation Center and Beijing Sullivan kindergarten. There were 10 children with a CI and 12 children using HA. In addition, the study included 22 children with normal hearing from Beijing Normal University kindergarten (13 boys and 9 girls). The teachers in the kindergarten assisted in acquiring parental consent. The children in the two groups were matched by age and gender in order to allow for an independent-samples t-test analysis. The results of the analysis showed that the difference between the mean ages of normal hearing children (54.41 ± 10.76 months) and children with a CI or HA (54.86 ± 11.97 months) was not statistically significant, *t*(42) = 0.132, *p* > 0.05. Of the 22 children with a CI or HA, 19 had over half a year of CI or HA experience and language rehabilitation. One had been using a HA for one month, and two who had been using a CI for 4 months. None of the children had an additional disability (such as blindness or autism). All children attended kindergarten from 8:00 am to 5:00 pm, 5 days a week, Monday to Friday, and received daily one hour, individual, auditory-oral therapy sessions. In addition, none of the parents had hearing impairments. There were six children living with their teachers because their parents worked in other cities. The participants with a CI or HA were selected by teachers who believed that they could understand the tasks. They all had prelingual deafness and did not know sign language. Mandarin was the children’s first language. The attributes of the children are shown in **Table 1**.

**Table 1:** Characteristics of participants in each group.

	Cochlear implants (CI)	Hearing aids (HI)	Normal
No. of children	10	12	22
Mean age (*SD*) (months)	52.00 (12.02)	57.25 (11.91)	54.41 (10.76)
Range of age (months)	30–70	41–84	34–77
Range of unaided-hearing loss (left/right)	100–115/85–115	60–103/50–108.5	//
Mean age of using CI or HA (*SD*) (months)	23.00 (16.08)	21.90 (10.70)	//
Ratio of males: females	3:7	10:2	13:9
Communication mode	Oral language	Oral language	Oral language

### Materials and Procedure

Color images of four basic emotions (happiness, sadness, anger, and fear) (Wang et al., 2011) were used in emotion-matching tasks and emotion-labeling tasks. Black and white images of four shapes (circle, square, rectangle, and triangle) were used as control tasks to measure children’s basic abilities of matching and labeling. The images were 7 cm by 9.5 cm.

#### Practice

Prior to the test trials, a color-matching task and an emotion-matching task, different from those used in the test trials, were used to ensure that the children understood both the concept of matching and the tasks. First, the experimenter or the teacher asked children to match the color. If a child did not successfully complete the color-matching task, the experimenter conducted more trials until the child correctly completed two consecutively. The children were then asked to match the images of emotional expressions. If a child could not complete the emotion-matching task, the experimenter or the teacher would instruct him or her the correct response and ask him or her to match the emotional expression again. Children who completed these two practice tasks could receive the formal test trials (matching task and labeling task).

#### Matching Test Task

**FIGURE 1:**
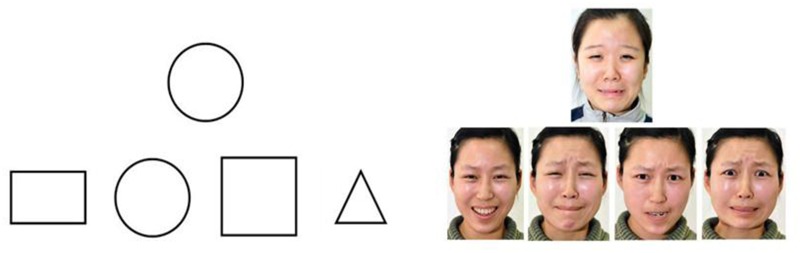
**The examples of materials for emotion and shape-matching tasks.** Selected from a series of Chinese emotional facial expressions collected by Guo Feng, Zheng Luo, Guangyuan Shi, and Chao Feng from department of psychology, Capital Normal University, Beijing.

This session included two tasks: emotion-matching task and shape-matching task. The shape-matching task was used to control for the presence of basic matching abilities. Children were asked to match the emotion or shape of a target stimulus at the top of a paper with one of the four choices presented at the bottom (see **Figure 1**). For example, “please match the same facial emotional expression”. The study included eight trials of emotion-matching. Two female and two male identity pairs completed eight trials of shape-matching. Each emotion and shape was used as the target stimulus twice. The position of the stimuli was balanced and the order in which they were presented was randomized. A correct response was given a score of 1 and an incorrect response was given a score of 0. The total scores for each of the emotion- and shape-matching tasks ranged from 0 to 8.

#### Labeling Test Task

This session also included two tasks: emotion-labeling task and shape-labeling task. The shape-labeling task was used to control for the presence of basic labeling abilities. Children were asked to point to the item that the experimenter asked for randomly, either an emotion (happiness, sadness, anger, and fear) (Wang et al., 2011) or a shape (circle, square, rectangle, and triangle) (control task). For example, “who is happy” was the child’s cue to point out the respective emotional expression that matched the label. The positions of the four facial expressions or shapes were counterbalanced. The order that men and women were presented in was also counterbalanced. A correct response was given a score of 1, and an incorrect response was given a score of 0. The total scores for the emotion- and shape-labeling tasks ranged from 0 to 8.

The paradigms of the matching and labeling task were used by Székely et al. (2011). In Ziv et al. (2013), the labeling task in the present study was named “pointing task”. The order of shape and emotion matching and labeling tasks was determined using a Latin-square design. SPSS 19.0 was used to analyze the data.

## Results

**FIGURE 2:**
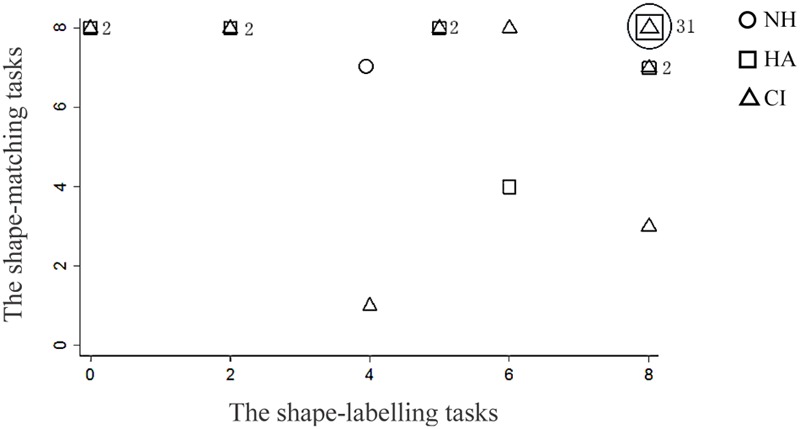
**Fractional distribution of the scores of the shape tasks.** Numbers in the graph represent the number of repeated data.

**FIGURE 3:**
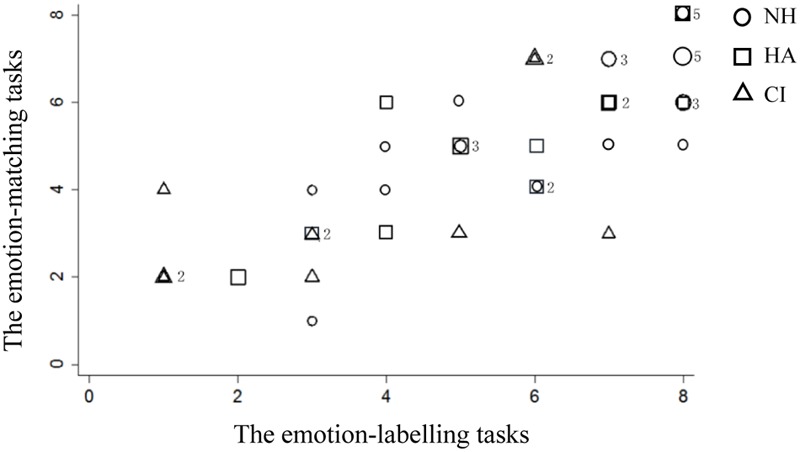
**Fractional distribution of the scores of the emotion tasks.** Numbers in the graph represent the number of repeated data.

Two scatter plots (see **Figures 2** and **3**) show the fractional distribution of different (shape and emotion) tasks and participants (normal hearing, CI and HA). **Figures 2** and **3** show that some participants received the same score, most notably for the shape tasks.

Following the fractional distribution, four homogeneity of variance tests were conducted. The scores of emotion-matching and emotion-labeling tasks showed homogeneity of variance [*F*(1,42) = 3.77, *p* > 0.05; *F*(1,42) = 2.65, *p* > 0.05]. However, the scores of shape-matching and shape-labeling tasks showed heterogeneity of variance [*F*(1,42) = 14.94, *p* < 0.05; *F*(1,42) = 26.96, *p* < 0.05].

We conducted a repeated measures ANOVA analysis utilizing the type of participant (normal/CI or HA) as a between-subject independent variable, and the type of task (matching/labeling) and type of stimuli (shapes/emotions) as within-subject independent variables. Because of the heterogeneity of variance, we used a Greenhouse–Geisser correction. It showed significant main effects for the type of participants, *F*(1,42) = 8.95, *p* < 0.01, η^2^ = 0.18, which indicated that hearing children did significantly better than children with a CI or HA. The test also indicated significance differences in the type of stimuli, *F*(1,42) = 63.38, *p* < 0.01, η^2^ = 0.60. The only significant interaction was between the type of task and the type of stimuli, *F*(1,42) = 11.43, *p* < 0.01, η^2^ = 0.21. Other interactions and the main effects for the type of task were not significant (*p*s > 0.05).

Based on a significant interaction between the type of task and the type of stimuli, we used a simple effect analysis. For both matching and labeling tasks, the scores using shape as stimuli were significantly higher than those using emotions as stimuli (*p* < 0.05). When the stimuli were emotions, the scores of matching tasks were significantly higher than those of the labeling tasks, *p* < 0.05. However, when the stimuli were shapes, no significant difference was present, *p* > 0.05.

Because of the four different emotion scores as dependent variables ranged from 0 to 2 only, nonparametric tests were used. Two Friedman tests indicated that for the matching and labeling tasks, significant differences existed among the four types of emotions (*p*s < 0.05) separately. Combined with the descriptive statistics shown in **Table 2**, the order of the four types of emotion scores from high to low was sadness, happiness, anger and fear for the labeling task and happiness, sadness, anger and fear for the matching task.

**Table 2:** Descriptive statistics for the labeling and matching task for each group (*M* (*SD)*).

Type of Tasks	Type of Shapes/Emotions	CI/HA	Normal	All
Shape-labeling	Circle	1.59 (0.73)	2.00 (0.00)	1.80 (0.55)
	Square	1.45 (0.80)	1.91 (0.43)	1.68 (0.67)
	Rectangle	1.41 (0.80)	1.91 (0.43)	1.66 (0.68)
	Triangle	1.64 (0.73)	2.00 (0.00)	1.82 (0.54)
	Total	6.09 (2.78)	7.82 (0.85)	6.95 (2.21)
Emotion-labeling	Happiness	1.23 (0.92)	1.77 (0.43)	1.50 (0.76)
	Sadness	1.55 (0.67)	1.68 (0.57)	1.61 (0.62)
	Anger	1.09 (0.87)	1.32 (0.65)	1.20 (0.77)
	Fear	0.82 (0.80)	1.05 (0.58)	0.93 (0.70)
	Total	4.68 (2.12)	5.82 (1.65)	5.25 (1.97)
Shape-matching	Circle	1.77 (0.53)	2.00 (0.00)	1.89 (0.39)
	Square	1.82 (0.40)	2.00 (0.00)	1.91 (0.29)
	Rectangle	1.77 (0.61)	1.95 (0.21)	1.86 (0.46)
	Triangle	1.82 (0.50)	2.00 (0.00)	1.91 (0.36)
	Total	7.18 (1.92)	7.95 (0.21)	7.57 (1.40)
Emotion-matching	Happiness	1.41 (0.73)	1.95 (0.21)	1.68 (0.60)
	Sadness	1.18 (0.85)	1.77 (0.61)	1.48 (0.79)
	Anger	1.36 (0.79)	1.36 (0.85)	1.36 (0.81)
	Fearful	1.00 (0.93)	1.50 (0.74)	1.25 (0.87)
	Total	4.95 (2.42)	6.59 (1.76)	5.73(2.25)

## Discussion

The results showed that children with a CI or HA were developmentally delayed in the performance of both emotion-labeling and emotion-matching. These present findings contradict the prior findings of Ziv et al. (2013) who found that for labeling and pointing tasks, there was no significant difference between children with a CI and hearing children. The two studies differ in two ways: the attributes of the participants and the experimental stimuli. In Ziv et al.’s (2013) study, the mean age of children with a CI was 6.6 years, whereas in the present study, the mean age of children with a CI or HA was 4.3 years. This is relevant because, according to Denham et al. (1990), FER development progresses with age, meaning differences in findings could possibly be attributed to the children being in different FER developmental phases. In addition, in Ziv et al.’s (2013) study, the mean age at implantation was 2.5 years, whereas in the present study, the nineteen children with a CI or HA had between half a year to 2 years of CI or HA experience and language rehabilitation, and the two children with CI and one child with a HA had less than half a year. During the early stage of rehabilitation, participants in the present study could not communicate with others fully and validly though they communicate orally. The present study also used the facial expressions of adult males and females, while Ziv et al.’s (2013) study used photographs of boys and girls who were the same age as the participants. Anastasi and Rhodes (2005) showed that it was easier for participants to interpret the emotional expressions of individuals in their own age group.

The relative difficulty of recognizing four different emotional expressions is similar between verbal and nonverbal tasks except for the order of happiness and sadness. These findings were inconsistent with the results of Székely et al. (2011). One important difference was the number of alternatives available to choose from during each trial. Székely et al. (2011) used only two alternatives during each trial of the matching task. In contrast, the present study used four alternatives. Another important difference was the different participants. All children in Székely et al. (2011) were 3-year-olds with normal hearing, while the participants in our experiment were children with normal hearing and children with a CI or HA that were between 30 and 84 months old.

The findings showed that both children with normal hearing and children with a CI or HA were most accurate when matching and labeling happy and sad faces, followed by angry and fearful faces. Vicari et al. (2000) found a similar rank in the four types of emotions. They reported that children who were between 5 to 10 years old consistently and regardless of age, recognized happiness and sadness, whereas the recognition of anger and fear improved with age. The findings of Ziv et al. (2013) indicated that happiness is the most difficult to recognize for the “pointing task”. This discrepancy is possibly due to the individual socio-culture experience and the complexity of facial expressions (Montirosso et al., 2010; Helen et al., 2015). Hence, it is hard to reach a universal conclusion on the developmental sequence of the FER (Vicari et al., 2000; Herba and Phillips, 2004; Helen et al., 2015).

The primary limitation of the present study was that we did not compare children with a CI to those with a HA due to the small sample size. However, Most and Aviner (2009) found no difference in the ability of children with a CI and those with HA to recognize facial expressions. An additional limitation of this study was that language ability was not measured.

To summarize, the recognition of facial expressions during verbal and nonverbal tasks was delayed in children with a CI or HA who were in early rehabilitation stage. For all participants, the emotion-labeling task was more difficult than the emotion-matching task. The relative difficulty of recognizing four different emotional expressions is similar between verbal and nonverbal tasks. The results of this study suggest that a future study of the rehabilitation process should be conducted to understand how it affects the development of FER in children with a CI or HA.

## Ethical Approval

All procedures performed in the study involving human participants were conducted in accordance with the ethical standards of the institutional and national research committee and with the 1964 Helsinki declaration and its later amendments or comparable ethical standards.

Written informed consent was obtained from all participants included in the study.

## Author Contributions

YW: Substantial contributions to the conception or design of the work. Analysis and interpretation of data for the work. Drafting the work. YS: Final approval of the version to be published. Revising it critically for important intellectual content. SY: Drafting the work. Acquisition of data.

## Conflict of Interest Statement

The authors declare that the research was conducted in the absence of any commercial or financial relationships that could be construed as a potential conflict of interest.
